# Extreme Situation Experienced by Dental Students of the Medical University of Silesia Due to the SARS-CoV-2 Epidemic during the First Lockdown

**DOI:** 10.3390/healthcare9111513

**Published:** 2021-11-05

**Authors:** Rafał Doniec, Sylwia Wójcik, Raul Valverde, Natalia Piaseczna, Szymon Sieciński, Konrad Duraj, Ewaryst Tkacz

**Affiliations:** 1 Department of Biosensors and Processing of Biomedical Signals, Faculty of Biomedical Engineering, Silesian University of Technology, Roosevelta 40, 41-800 Zabrze, Poland; natalia.piaseczna@polsl.pl (N.P.); szymon.siecinski@polsl.pl (S.S.); konrad.duraj@polsl.pl (K.D.); etkacz@polsl.pl (E.T.); 2 Dental Surgery Department at the Chair and Clinic of Maxillofacial Surgery and Dental Surgery, School of Medicine with the Division of Dentistry in Zabrze, Medical University of Silesia in Katowice, Pl. Akademicki 17, 41-902 Bytom, Poland; sylwiawojcik6@poczta.onet.pl; 3 John Molson School of Business, Concordia University, 1450 Guy Str., S-MB 11311, Montreal, QC H3H 0A1, Canada; rvalverde@jmsb.concordia.ca

**Keywords:** COVID-19, stress level, university students, education

## Abstract

The pandemic declared in many countries in 2020 due to COVID-19 led to the freezing of economies and the introduction of distance learning in both schools and universities. This unusual situation has affected the mental state of citizens, which has the potential to lead to the development of post-traumatic stress and depression. This study aimed to assess the level of stress in dental students in the context of the outbreak of the SARS-CoV-2 virus pandemic. A survey on the PSS-10 scale was prepared to measure the level of perceived stress. The study included 164 dental students at the Faculty of Medical Sciences of the Medical University of Silesia in Katowice, Poland. The results showed the impact of COVID-19 on the stress of students, with 67.7% reporting high levels of stress. The study also revealed that stress was higher among older female students. This paper recommends that the university provide more intensive psychological care as psychological first aid strategies in epidemics or natural disasters and to consider telemedicine in order to deliver services due to the limitations of the pandemic.

## 1. Introduction

On 12 December 2019, the first cases of the SARS-CoV-2 virus appeared in Wuhan, China. This virus is responsible for the development of severe respiratory distress syndrome (SARS) [1,2,3]. Human-to-human transmission of the virus occurs through direct or indirect routes. The direct route is associated with droplet transmission, while the indirect route is associated with contact with contaminated surfaces. The entry sites are the nose, mouth, and eyes [4,5,6,7]. Therefore, the SARS-CoV2 virus is a biological risk factor to which dental practice staff are inherently exposed: from doctors, through assistants, to patients.

Until the pandemic, routine dental procedures did not refer to the prevention of pathogen transmission by airborne droplets. For that reason, new and additional methods for prevention of spreading SARS-CoV-2 had to be introduced into the day-to-day work of dentists. These methods involve the use of additional personal protective equipment in the form of face masks, goggles, or special uniforms, as well as new methods for the decontamination of the office environment [5].

In most countries affected by the pandemic, new measures were introduced to deal with the situation, including, among others, the postponement of unnecessary dental treatments, and therefore dental treatments were discontinued and limited only to emergency cases [6,8]. The healthcare community, including medical, dental, pharmacy, and nursing students, has become the line of first contact with the people infected with the SARS-CoV-2 virus [9].

During the pandemic, many professions, including dental practice, have undergone major changes, particularly with regard to protection measures. The impact of the pandemic on dental professionals has been confirmed by research conducted worldwide. These studies covered the pandemic’s impact on various aspects of life, including, among others, financial and professional status, nutrition, and changes in family dynamics [10,11,12]. Mental health of dental workers (dentists, dental students, and support personnel) during the pandemic has also become a subject of many studies. These studies include the effects of lockdown on depression and the level of perceived stress [13,14,15].

Fear and anxiety experienced by dental students can be induced by many different factors [16,17,18], one of them being the transmission of infectious diseases. Since this virus is mostly transmitted by droplets, and dental procedures generate aerosols, dental students may feel stressed about returning to clinical classes while the COVID-19 pandemic continues [19].

It has been noticed that even young people are concerned about getting infected with SARS-CoV-2 due to the possible side effects. Due to the specificity of the dentist profession (mainly clinical practice), dental students may suffer even more in the future due to the lack of training in manual skills [20,21].

Despite the COVID-19 pandemic, dental studies are considered very stressful because, during the curriculum, students must face their practical skills and the ability to establish contact with the patient. An essential element that cannot be learned without direct contact with the patient is the ability to relieve stress in the patient, which often results from empathy [16,17,18,22]. Dental studies require full-time dedication, especially at the beginning of the curriculum. Freshmen students need to develop practical skills (which takes a significant amount of time) to then participate in patients’ visits. The students are pressured with the responsibility for the patient’s treatment and possible emerging complications [16]. The factors mentioned earlier influence the higher stress level among dental students comparing to the general population [23,24,25].

In their study, Abu-Ghazaleh et al. showed an increasing amount of anxiety in dental students at the University of Jordan over the years of training; over the year of studies, the awareness of the responsibility developed and caused increased stress levels [26]. The pressure on dental students is replaced by stress resulting from the need to cope with professional duties and full-time clinical work and to simultaneously carry out continuing postgraduate education [27].

Stress and anxiety affect many aspects of the work of a dental practician, which in effect has an impact on the entire community [28]. Accordingly, it is imperative to determine the stress level of dental students as well as to consider stress management measures [29].

According to Davidovich et al., junior students experience a significantly higher level of stress than experienced professionals. The newcomers rely on their knowledge, which is theoretical and may not yet be sufficient. Moreover, they did not yet receive enough practical training, which causes performance anxiety [29].

Failure to adapt the requirements to the individual’s abilities may cause stress, which is a physical, mental, or emotional reaction to change [30]. Stress has become a significant problem of the modern world as it can lead to cognitive impairment, depression, and even cardiovascular diseases [31]. Stress can also affect our daily attitudes and our ability to be involved in work [32,33,34].

The methods of coping commonly used by dental students are various relaxation exercises as well as emotional support from other people [35,36]. Dental schools also offer ways to reduce one’s stress levels, including stress management courses, relaxation techniques, and counselling [37,38].

Despite all the enforced anti-COVID-19, medical students must acquire some skills through practical contact classes. Only in this way are they able to acquire the competences needed to work in the profession for which they are being prepared and educated [39].

### Objective

Referring to the issues discussed above regarding, among others, fear of possible infection with the SARS-CoV-2, reduced number of practical classes, difficulties in acquiring theoretical knowledge, and fear for the health of loved ones, we in this paper aimed to assess the level of stress in dental students in relation to the outbreak of the SARS-CoV-2 virus pandemic.

## 2. Materials and Methods

The study included 164 of 225 dentistry students at the Faculty of Medical Sciences of the Medical University of Silesia in Katowice, Poland.

Thirty first-year students (23 women, 7 men), 19 second-year students (13 women and 6 men), 27 third-year students (22 women and 5 men), 32 fourth-year students (26 women and 6 men), and 56 fifth-year students (35 women and 21 men) participated in the study. The median age of the students was 23 years. The students voluntarily completed the survey, which was available online. The response rate was 0.80. A total of 180 dental students completed the questionnaire. However, due to procedural errors (lack of all responses), 16 questionnaires were not considered in the statistical analysis.

The link to the questionnaire was placed on the website of the Department of Conservative Dentistry with Endodontics of the Medical University of Silesia (available at www.stom.zachowawcza.sum.edu.pl (accessed on 24 April 2020)) and sent by e-mail to the representatives of all years of the study (Years 1–5) with a request to be sent to students. General information about the survey and the study was provided in this e-mail. The consent to participate in the study was part of the questionnaire.

The questionnaire was placed on the e-learning platform of the Medical University of Silesia. The platform is based on the Moodle system, which is a free remote learning management system (LMS) under an open-source license [40]. The system has enormous support from the broadly understood community dealing with distance learning topics. In its present form, the platform was launched in the academic year 2017/2018. The current version of the system was 3.7 in the latest stable update.

The system is based on university server resources, using a virtual multiprocessor environment. This is supported by a stable university computer network. The e-learning platform is available to users continuously, 24 h a day, 7 days a week. Currently, the system covers all the university’s faculties, along with most research and teaching units. Currently, there are more than 15,000 active accounts in the system and more than 7500 unique users visit the system every day and actively work with it.

The survey system is also placed on university servers within the information portal with the installed extension, which enables independent design and publication of survey forms. The server is available to users continuously, 24 h a day, 7 days a week.

The Perceived Stress Scale (PSS-10) was used in the survey. This scale is a modified version of the Perceived Stress Scale developed by Cohen [41]. It was used to assess the intensity of stress related to one’s own life situation over a month. The authors conducted a survey using the above scale among students between 10 April 2020 and 24 April 2020, during the SARS-CoV-2 virus pandemic state of emergency announced in Poland, as well as globally.

PSS-10 contains 10 questions related to a variety of subjective feelings about personal problems and events, behaviors, and ways of coping. The PSS-10 scale has been used to test the mental status of adults. It is mainly used as a self-assessment method and may also be used as a screening tool to identify people eligible for psychological or medical assistance [42]. The respondents gave their answers by entering the correct digit, that is, 0 meaning “never”, 1 “almost never”, 2 “sometimes”, 3 “quite often”, and 4 “very often”. Before the general indicator of the intensity of perceived stress is calculated, a change in the scores should be made in the answers to the positively formulated questions, that is, 4, 5, 7, and 8, according to the rule: 0 = 4, 1 = 3, 3 = 1, 4 = 0. The overall score on the scale is the sum of all points, the theoretical distribution of which ranges from 0 to 40. The higher the score, the greater the perceived stress. To facilitate the interpretation of the results, we interpreted the total sum score obtained according to the properties characterizing the sten scale. The study was designed so that the dependent variable Y represents the Perceived Stress Scale (PSS), which is the most commonly used psychological tool to measure the perception of stress. It is a measure of the extent to which life situations are judged to be stressful. For example, Cohen et al. (1988) show correlations with PSS and stress measures, measures of self-assessment of health and health services, measures of health behavior, smoking status, and behavior in search of help. PSS scores range from 0 to 4 and measure how often participants felt different variables (e.g., 0 = never, 1 = almost never, 2 = sometimes, 3 = quite often, and 4 = very often for X1 = voltage). It is a measure of the extent to which life situations are judged to be stressful. The items are designed to show how unpredictable, uncontrolled, and overloaded respondents find their lives.

According to the PSS-10 scale calculation instruction, scores ranging from 1 to 4 stens (0–13 points on the scale) are treated as low stress levels. Scores within 5–6 stens (14–19 points on the scale) correspond to the average stress level. Finally, 7–10 stens (20–40 points on the scale) indicate a high stress level [43].

The consent to conduct a survey using, among others, the above-described scale in various groups of respondents was issued by the Bioethical Committee of the Medical University of Silesia in Katowice, KNW/0022/KB1/79/18. Students started the study by signing a consent form.

### Statistical Analysis

Statistical analysis was performed using Statistica version 9.0 (StatSoft, Inc., Tulsa, OK, USA) and Microsoft Excel spreadsheet (Microsoft Corporation, Redmond, WA, USA). The Mann–Whitney test was used to assess the significance of differences in the values for the PSS-10 point and the sten scale from the point of view of the gender of the respondents and the year of study of the students.

Assessment of the significance of differences in the PSS-10 point and the sten scale with respect to subsequent years of study and the gender of respondents was carried out using the Kruskal–Wallis test. To test the correlation between the stress level of the respondents and age, we used the Spearman correlation test.

A multiple variable linear regression model was used to develop a stress model that can be applied to stress estimation based on PSS-10 variables. Independent variables were selected using a stepwise regression that included a set of regression models in which the choice of predictive variables was carried out using a sequence of F-tests. Once the final data set and independent variables were chosen, a multiple variable linear regression was performed, and a stress model was developed that can be applied to estimate stress levels.

## 3. Results

To confirm the reliability of the PSS-10 questionnaire, we calculated Cronbach’s *α* for the selected group of respondents, which was 0.86 for students (*α* = 0.85 for women; *α* = 0.86 for men). The most difficult question was Question 5 (“How often in the last month have you felt that things were going your way?”). Its elimination could lead to an increase in Cronbach’s coefficient, but it would not be a statistically significant increase.

**Figure 1:**
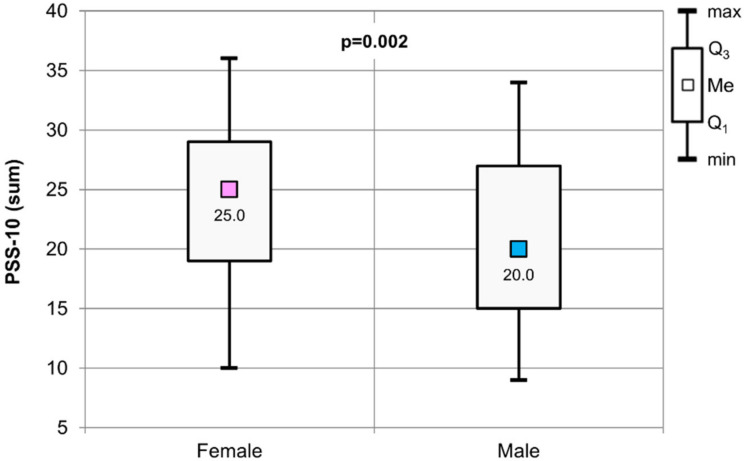
The results of the Mann–Whitney test used to assess the significance of difference values for the PSS-10 (sum) scale in relation to the gender of the respondents.

The results of the Mann–Whitney test used to assess the significance of the differences in values for the PSS-10 scale from the point of view of gender of the surveyed students are presented in Figure 1. Women were characterized with a significantly higher perceived stress level than men (*p* = 0.002).

The median value of the PSS-10 scale obtained for female students was 25, and for male students, 20. The highest number of points on the scale was obtained for female students (36). The highest result on the PSS-10 scale in male students was 34. The lowest point result was 10 and 9 for female and male students, respectively.

**Figure 2:**
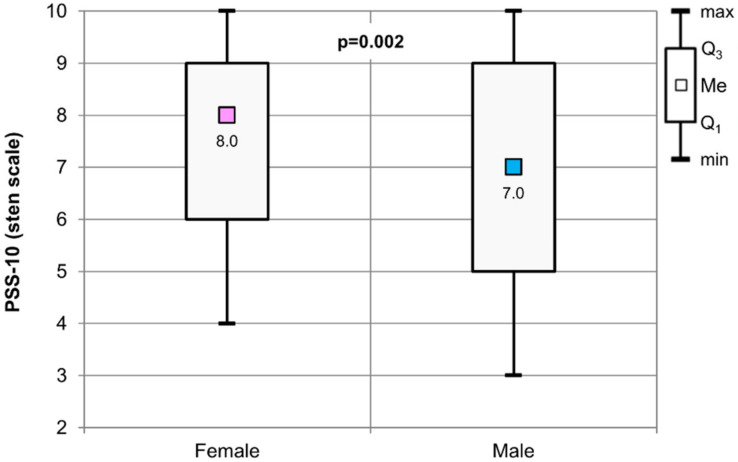
The results of the Mann–Whitney test used to assess the significance of the difference values for the PSS-10 (sten scale) in relation to the gender of the respondents.

Analyzing the results obtained in terms of interpretation on the sten scale, we found statistically significant differences between female and male students (*p* = 0.002). The median of the sten was 8 for female students and 7 for male students. The highest value obtained on the sten scale for both women and men was 10, while the lowest was 4 in female students and 3 in male students (Figure 2).

Table 1 shows the distribution of respondents according to whether they belonged to the appropriate group of stens. A total of the 73.9% of female students and 51.1% of the male students were characterized by a high level of stress, as evidenced by the results obtained between 7 and 10 stens. To confirm the hypothesis that the gender difference in the sten scale was significant, we applied the χ^2^ test of independence. The result of the χ^2^ test indicated the significance of examined dependence (χ^2^ = 12,884, *p* = 0.045).

The Spearman correlation test showed a positive statistically significant correlation in the group of female students between the age of the respondents and the obtained values of the PSS-10 scale (*p* = 0.007) and between age and sten values (*p* = 0.009). The results showed that stress level was related to the age of female students (see Table 2 and Table 3).

**Figure 3:**
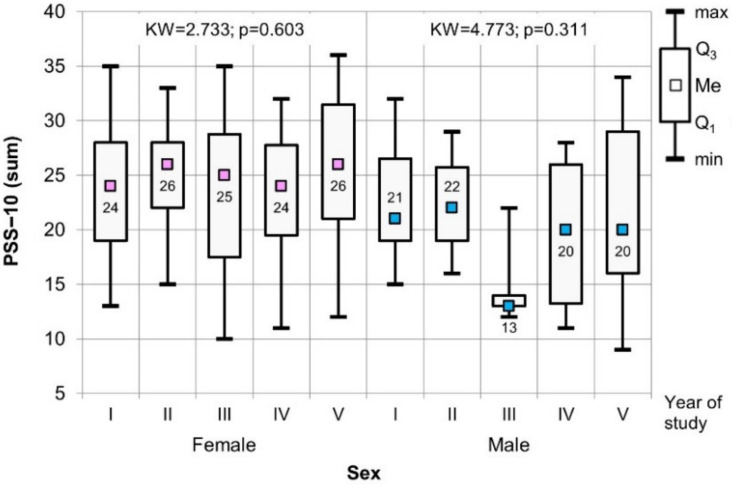
Results of the Kruskal–Wallis test used to assess the significance of difference values for the PSS-10 (sum) scale in relation to gender of respondents and year of study.

**Figure 4:**
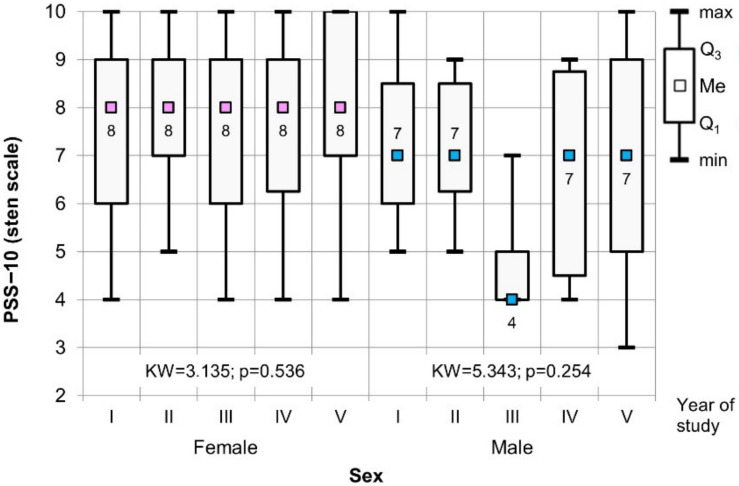
The results of the Kruskal–Wallis test used to assess the significance of the difference values for PSS-10 (sten scale) in relation to the gender of the respondents and the year of study.

The comparative analysis, considering the gender of the respondents and the year of study using the Kruskal–Wallis test, did not show statistically significant differences between the individual years of study, both in the case of the PSS-10 and the sten scale (Figure 3 and Figure 4).

A simple linear regression model was developed for each independent variable for the PSS-10, as indicated in Table 4. We performed a stepwise regression starting with the independent variable with the highest F-value. The remaining independent variables were added one at a time in the order of the highest F-value. Independent variables were added when adding them improved the model. The improvement of the model was determined by increasing the R^2^ value.

The 10 independent variables improved the accuracy of the model, and therefore none of them were removed from the final equation (see Table 5).

## 4. Discussion

One of the methods to prevent the spread of airborne infectious diseases is the isolation (quarantine) of infected people and those who meet infected people. However, such isolation, carried out for the benefit of the public, may have disastrous consequences for the individual in the form of psychological, emotional, and financial problems [44]. Due to the specificity of transmission of the SARS-CoV-2 virus, which can cause asymptomatic cases [1], many countries, including Poland, have introduced amass isolation of society, which is also associated with the closure of schools and universities. According to the regulations of the Minister of Health from 11 March 2020, all classes should be conducted using distance learning methods and techniques [45].

During the coronavirus pandemic, students have been exposed to many stressful situations, such as disturbances during academic activities and limitations of social and family contacts [39]. Limiting academic classes for dental students to only theoretical online classes can be very worrying due to the specifics of the study program, which is largely based on practical classes [46].

Kharma et al. conducted a survey among 315 dental students from Al-Farabi Private College (Jeddah, KSA) on the assessment of anxiety and stress before returning to clinical classes after lockdown. A total of 85% of the participants experienced stress and anxiety before returning to class, 58% of the respondents felt a high level of stress due to the necessity of contact with people outside the academic community, 75% of the students supported the safety procedures while admitting patients introduced by the college, and 43% of the respondents indicated remote classes as the only acceptable form of theoretical classes [19].

In the study by Jum’ah et al., fear related to the high risk of SARS-CoV-2 infection was also observed. According to this study, 92.7% of dental students felt fear of infection, 78.1% of the participants experienced the anxiety of transferring the disease to family members, and 56.1% of the students confirmed that fear of infection caused stress that adversely affected the performance of dental procedures during clinical classes [39].

In our study, female students were characterized by a significantly higher level of stress than male students. Furthermore, a positive correlation was observed between the age of the women and the level of perceived stress. The older the women, the more stress they felt.

Dental students who participated in the study by Hung et al. faced similar problems. Many of them (32%) were concerned about their emotional state. A total of 29% of the participants felt they could not control important things in their lives, while 24.3% of them always or often felt angry due to lack of control, and 37% of the students experienced stress. Almost half of the respondents felt anxious about the state of the pandemic, and 8.3% suffered from depression [47].

Leon-Manco et al. conducted a study on a group of 2036 dentists and 1433 female dental students using the PSS-15 scale. They used descriptive, both two-dimensional and multi-dimensional linear regression analysis to observe factors related to the perception of stress. Mean PSS-14 score was 24.76 (±11.76). Hierarchical regression models turned out to change significantly along with PSS-14 results, specifically with income levels during compulsory social isolation, having elderly people in care during compulsory social isolation, self-assessment of the level of anxiety related to COVID-19, self-assessment of health, and coffee consumption during the period of compulsory social isolation. The authors of these studies emphasized that the pandemic affected the personal, social, and professional lives of dentists and dental students. Its impact on the mental health of this population is also undeniable, especially when perceived stress is taken into account. Consequently, mental health monitoring strategies and systems need to be put in place for this population [12].

The results of our study showed that a significant proportion of surveyed students (i.e., 73.9% female and 51.1% male) experienced a high level of stress determined by values between 7 and 10 on the PSS-10 scale. The factors that influenced stress to the greatest extent were tension, lack of self-control, and lack of resilience.

Statistically significant differences in the level of perceived stress between men and women were also observed among dental students in Saudi Arabia. Female students were significantly more stressed and experienced higher levels of anxiety and depression. These differences were not observed between years of study, as in our research [48].

The fact of women experiencing higher levels of stress and anxiety is also confirmed in [14,49,50,51,52]. The psychological differences between the genders are that women are more likely to express emotions [52]. Female students, especially those in their second and fifth years, experienced higher stress values than their peers. Third- and fourth-year female students also demonstrated higher stress values when compared to men, but these were only on the border of statistical significance. Similar results were also reported by Saddki et al. and other authors [53,54,55].

The higher levels of perceived stress among women are likely caused by their higher susceptibility to physical and emotional discomfort [56]. Moreover, the influence of marital status on the level of perceived stress was noticed. Unmarried students (and similarly the ones who were separated/widowed/divorced) showed higher stress levels in terms of factors affecting their future than married students. They were less confident in the decisions they made [57].

Another aspect worth considering is the impact of the COVID-19 pandemic on the education system and thus the level of stress felt by students as a result of new learning conditions. Iosif et al. conducted a cross-sectional study of 878 dental students who assessed their perceptions of the psychological and educational impact of the pandemic period. The authors found a strong psychological influence among the respondents—the level of stress was perceived as high and very high (33.83%, n = 297; 28.59%, n = 251, respectively). Similarly, strong and very high anxiety feelings were noted (26.54%, n = 233; 24.26%, n = 213, respectively). A very high educational impact was noted in terms of acquiring practical skills (48.52%, n = 426) and future career prospects (38.95%, n = 342). Theoretical online learning ability was generally low (37.93%, n = 333). The majority of students rated the effectiveness of lecturers in online courses as neutral (41.12%, n = 361). New dentistry curricula will need to be developed, taking into account the dynamics of the pandemic and its strong impact on students’ livelihoods in order to improve both their well-being and the sustainability of dental education.

It is worth noticing that the COVID-19 pandemic has shaken the entire planet, forcing the entire world’s population to reconsider all their relationship and interaction habits to avoid the epidemic from spreading. COVID-19 has also left its mark on education and grading methods. Changes in teaching methods that occurred in institutions had to be made suddenly, affecting the stress levels of staff and students [50]. The level of stress of dentistry students in other countries can be implied using the scope of the study, but it should be noted that the subjects were concentrated in a specific geographic area and the results cannot be generalized to the entire globe.

### 4.1. Limitation of the Study

The limitations of the study include the study group drawn from only one university, the statistical analysis that concentrated on gender differences, and the year of study in students, and there was a predominance of female subjects in the study group. The gender bias in the study group is typical in academic medical programs in Poland [58]. However, the study group should be more heterogenous and with an equal group of male and female subjects in future studies. Moreover, the data related to stress before the pandemic were not available.

### 4.2. Practical Application

To reduce the levels of stress in society, the authorities must implement psychological first aid strategies in epidemics or natural disasters, and ultimately, the role of telemedicine must become more significant in this case. The COVID-19 pandemic, which has affected the entire world, should influence the development of methods that allow comprehensive epidemiological surveillance and screening of people who may develop mental disorders [59].

A good strategy to reduce stress levels is to train individuals to reduce tension, have more controls of themselves, and be more resilient. These can include strategies that have been shown to be effective, such as biofeedback [60], mindfulness [61], and resilience training [62].

## 5. Conclusions

This article presents the results of the first academic study conducted at the Medical University of Silesia on the experiences of students in social isolation in early 2020. The survey showed the effectiveness of the PSS-10 scale in screening the level of perceived stress. Regarding female students, the older they were, the higher the level of stress they perceived themselves to have endured. Finally, more than half of the surveyed respondents (67.7%) felt a high level of stress related to the outbreak of the SARS-CoV-2 pandemic. Tension, lack of self-control, and lack of resilience were the most influential factors in the stress levels in the people being surveyed. This analysis indicates that the individual can potentially be in control of his or her own stress level. Stepwise regression analysis also confirmed that all 10 variables on the PSS-10 scale were relevant to explaining stress levels in people. The survey includes follow-up questions on respondents’ mental health, such as anger, irritation, tension, or lack of self-control, which are the main contributors to mental health problems.

The primary purpose of the survey was to investigate the impact of the pandemic on mental health. The unique fact is that the pandemic is a memorable period. The results from such a study are non-referential, at least until the next pandemic.

To achieve more accurate results in the future, we consider including additional questions for the survey combined with measurements from blood pressure, heart rate, or any other physiological signal. For the analysis, we hope to involve the use of deep learning methods to achieve higher accuracy and make text responses more arbitrary.

Social distancing and tightening of sanitary standards related to the COVID-19 pandemic have increased the stress among many social groups, including dental students. The situation has beyond doubt affected the world and will have a significant impact on the development of stress-coping techniques, which, as everything indicates, will be more and more needed.

## Figures and Tables

**Table 1:** The distribution of respondents in different sten scales.

Sten Scale	Female Students	Male Students	Total
1–4	7 (5.9%)	9 (20.0%)	16 (9.8%)
5–6	24 (20.2%)	13 (28.9%)	37 (22.6%)
7–10	88 (73.9%)	23 (51.1%)	111 (67.7%)
Total	119	45	164

**Table 2:** Results of the Spearman correlation test—correlation between age and PSS-10.

	Female Students	Male Students
rs	0.2257	−0.0616
*p*	0.0068	0.3439

rs—Spearman correlation coefficient.

**Table 3:** Results of the Spearman correlation test—correlation between age and sten scale.

	Female Students	Male Students
rs	0.2174	−0.0192
*p*	0.0088	0.4501

rs—Spearman correlation coefficient.

**Table 4:** F-value and coefficient of determination for each independent variable.

Variables	F-Value	Coefficient of Determination (R^2^)
Level of tension (X1)	269.02	0.61
Level of lack of self-control (X2)	247.51	0.59
Level of lack of resilience (X3)	218.91	0.56
Level of anger (X4)	186.33	0.52
Level of ability to cope with uncertainty (X5)	179.02	0.51
Level of lack of ability to cope with responsibilities (X6)	146.52	0.46
Level of ability to cope with irritability (X7)	119.52	0.41
Lack of ability to deal with personal problems (X8)	63.62	0.27
Level of sensation that things were going your way (X9)	51.37	0.23
Level of feeling that everything was going well (X10)	45.72	0.26

**Table 5:** Multi-regression for stress estimation for PSS-10.

Y = 0.95 + 0.33 * X1 + 0.29 * X2 + 0.3 * X3 + 0.3 * X4 + 0.26 * X5 + 0.25 * X6 + 0.27 * X7 + 0.22 * X8 + 0.27 * X9 + 0.27 * X10	**Coefficient of Determination (R^2^) 0.97**

## Data Availability

The data presented in this study are available on request from the corresponding author.

## Abbreviations

COVID-19	Coronavirus disease-2019
DASS-21	Depression, anxiety, and stress scale
MERS	Middle East Respiratory Syndrome
OK	Oklahoma
PSQ	Pandemic Stress Questionnaire
PSS-10	Perceived Stress Scale
SARS	Severe acute respiratory syndrome
USA	United States of America
WA	Washington
